# Survival of multiple arterial grafting in diabetic populations: a 20-year national experience

**DOI:** 10.1093/ejcts/ezad091

**Published:** 2023-03-16

**Authors:** Justin Ren, Colin Royse, David H Tian, Aashray Gupta, Alistair Royse

**Affiliations:** Department of Surgery, The University of Melbourne, Melbourne, VIC, Australia; Department of Surgery, Royal Melbourne Hospital, Melbourne, VIC, Australia; Department of Surgery, Outcomes Research Consortium, Cleveland Clinic, Cleveland, OH, USA; Department of Surgery, The University of Melbourne, Melbourne, VIC, Australia; Department of Anesthesia, Westmead Hospital, Sydney, NSW, Australia; Department of Surgery, University of Adelaide, Adelaide, SA, Australia; Department of Surgery, Gold Coast University Hospital, Southport, QLD, Australia; Department of Surgery, The University of Melbourne, Melbourne, VIC, Australia; Department of Surgery, Royal Melbourne Hospital, Melbourne, VIC, Australia

**Keywords:** Coronary artery bypass grafting, Multiple arterial grafting, Diabetes mellitus, Radial artery, Bilateral internal mammary artery, Total arterial revascularization

## Abstract

**OBJECTIVES:**

Diabetics may have diminished survival after coronary artery bypass grafting even with multiple arterial revascularization. We compared multi-arterial versus single-arterial grafting (SAG) survival in diabetic and non-diabetic patients undergoing primary isolated bypass surgery.

**METHODS:**

This is a retrospective analysis of the Australian and New Zealand Society of Cardiac-Thoracic Surgical Database from June 2001 to January 2020. Patients were classified as having either single or multiple arterial grafting irrespective of the number of venous grafts. The end points were long-term all-cause mortality and 30-day clinical outcomes, which was compared in 1:1 propensity score-matched patients. Cox regression model was used to assess interactions between diabetes and the treatment effect of multi-arterial grafting, reported as hazard ratios (HRs) and confidence intervals (CIs). Short-term outcomes were compared with McNemar’s paired *t*-test.

**RESULTS:**

From 69 624 patients, matching generated 17 474 non-diabetic and 10 989 diabetic patient pairs. At a median [interquartile range] of 5.9 [3.2–9.6] years postoperative, mortality was significantly lower after multi-arterial grafting for both diabetic (HR, 0.83; 95% CI, 0.76–0.90, *P* < 0.001) and non-diabetic (HR, 0.88; 95% CI, 0.82–0.95; *P* < 0.001) cohorts than SAG. The incidence of 30-day myocardial infarction was significantly higher in single than multiple arterial grafting for both cohorts (diabetic, *P* = 0.029; non-diabetic, *P* < 0.001). The interaction analysis suggested an insignificant effect of diabetes (*P* = 0.55) on the observed survival advantage. Further stratification by diabetic management generated consistent results.

**CONCLUSIONS:**

Multi-arterial grafting was associated with improved overall survival compared to SAG for both non-diabetic and diabetic patients.

**Figure:**
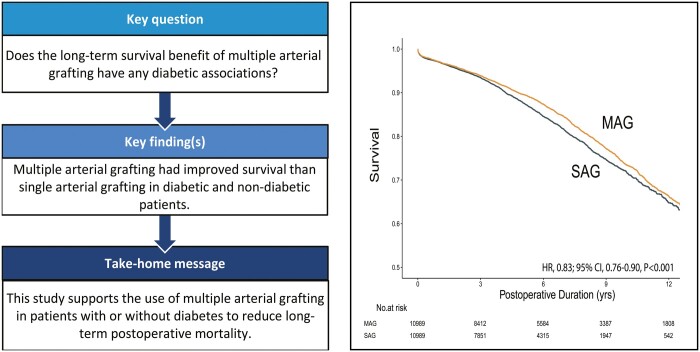

## INTRODUCTION

Diabetes mellitus (DM) has become increasingly prevalent [1] as one of the major risk factors for coronary artery disease, which increases the anatomic disease severity and postoperative mortality [2] in patients undergoing coronary revascularization. Coronary artery bypass grafting (CABG) is shown to have superior outcomes compared with percutaneous coronary intervention for diabetic patients with multivessel coronary artery disease [3–5].

Conventional technique involves the left internal mammary artery and supplementary saphenous vein grafts (SVG). However, multiple arterial revascularization has gained popularity due to the common late progressive atherosclerosis of SVG. Recent international guidelines recommend the use of a second arterial conduit in patients without a contraindication [6]. Strong evidence has reported significant relative reduction of mortality and myocardial infarction (MI) associated with multiple arterial grafting (MAG) [7, 8], but uncertainty exists over whether the benefits of MAG outweigh the operative risks in the diabetic populations.

We therefore sought to compare the long-term survival of patients undergoing MAG versus single-arterial grafting (SAG) in diabetic and non-diabetic cohorts in Australian & New Zealand Society of Cardiothoracic Surgeons (ANZSCTS) National Database.

## METHODS

### Ethics statement

The Melbourne Health Institutional Review Board (# 2011.164) has approved this study with an official waiver for individual patient consent.

### Study design

This is a retrospective observational cohort study that identified all adult patients who underwent primary isolated CABG with ≥2 grafts at 59 cardiac centres between June 2001 and January 2020 from the ANZSCTS registry. This national database prospectively records patient demographic, risk factors and procedural details checked regularly and validated through multiple administrative linkages, including to the National Death Index. Patients who had reoperations, concomitant or previous cardiac surgery procedures, single-graft and no arterial grafting were excluded. Complete case analyses were conducted.

### Patient selection

MAG was defined as receiving at least 2 arterial grafts with or without supplementary SVG, whereas SAG patients had only 1 arterial graft and at least 1 supplementary SVG. No further distinctions were made with regards to preoperative and intraoperative characteristics. The choice of graft configurations was by surgeon preference. All procedures and medical management were at the discretion of each centre but no considerable change in practice was reported throughout the study period.

A total of 36 preoperative and intraoperative baseline covariates were collected for both groups, including age, sex, body mass index, creatinine level, hypertension, hypercholesterolaemia, DM, smoking history, dialysis, arrhythmia, cerebrovascular event, peripheral vascular disease, chronic obstructive lung disease, MI, left ventricular ejection fraction, congestive heart failure, New York Heart Association classification, left main disease, number of grafts, number of diseased vessels, operative status, on-pump and minimally invasive surgery.

### Study outcomes

The primary end point was all-cause mortality reported as time-to-event measured from the date of the index operation to the longest follow-up. Administrative linkage had population coverage for death from a Federal Government agency with completeness of follow-up measured by follow-up index [9]. Secondary end points included 30-day mortality and 30-day readmission to the hospital due to a composite of MI, deep sternal wound infection (DSWI), arrhythmia, congestive heart failure or recurrent angina. Each component was then individually analysed. Readmission excluded admission to emergency, short-stay wards or planned transfer to rehabilitation facility.

For further evaluation, the diabetic population was stratified by degree of disease severity. The 3 subgroups were no medical intervention (no treatment or dietary control only), oral medication and insulin dependent patients, in increasing order of severity. Classifications were determined by the most aggressive diabetes control therapy at the time of surgery. Patients with insulin usage may receive other treatments simultaneously.

### Statistical analysis

A complete case retrospective analysis was performed with categorical variables reported as frequency counts and percentages, and continuous variables reported as median [interquartile range] or mean ± standard deviations (SDs) after assessment of normality.

Propensity score matching (PSM) was used to minimize potential selection bias by controlling for imbalanced baseline characteristics. Using generalized linear regression, the propensity score estimation algorithm incorporated all 36 variables from Table 1 to balance covariates between MAG and SAG cohorts. Non-replacement one-to-one greedy matching with calliper width of 0.2 SD of the logit of the propensity score was performed separately for the diabetic and non-diabetic cohorts. Absolute standardized mean differences were evaluated before and after PSM for balance diagnostics; a difference value of <0.1 generally indicated balance between patient groups [10].

**Table 1:** Cohort demographics from the Australian & New Zealand Society of Cardiothoracic Surgeons database before matching

Variables	MAG group (*n* = 39,478)	SAG Group (*n* = 30 146)	Diabetic group (*n* = 25 751)	Non-diabetic group (*n* = 43 873)
Age	65.0 ± 10.2	67.1 ± 10.0	65.6 ± 9.7	66.0 ± 10.4
Male	32 559 (82.5)	24 046 (79.8)	20 015 (63.6)	36 590 (83.4)
Body mass index	29.0 ± 5.2	28.9 ± 5.4	30.1 ± 5.6	28.3 ± 4.9
Creatinine (μmol/l)	95.0 ± 60.2	105.2 ± 94.7	108.4 ± 97.8	94.1 ± 61.4
Hypertension	30 718 (77.8)	24 669 (81.8)	22 642 (87.9)	32 745 (74.6)
Hypercholesterolemia	31 932 (80.9)	24 291 (80.6)	22 384 (86.9)	33 839 (77.1)
Diabetes mellitus	13 794 (34.9)	11 957 (39.7)	25 751 (100)	0 (0.0)
No treatment	256 (0.6)	227 (0.8)	483 (1.9)	0 (0.0)
Diet control	1900 (4.8)	1336 (4.4)	3236 (12.6)	0 (0.0)
Oral therapy	7823 (19.8)	6526 (21.6)	14 349 (55.7)	0 (0.0)
Insulin dependent	3815 (9.7)	3868 (12.8)	7683 (29.8)	0 (0.0)
Smoking history	25 360 (64.2)	19 483 (64.6)	16 671 (64.7)	28 172 (64.2)
Dialysis	265 (0.7)	717 (2.4)	647 (2.5)	335 (0.8)
Arrhythmia	3132 (7.9)	3062 (10.2)	2329 (9.0)	3865 (8.8)
Cerebrovascular event	3615 (9.2)	3267 (10.8)	3060 (11.9)	3822 (8.7)
Peripheral vascular disease	3909 (9.9)	3357 (11.1)	3731 (14.5)	3535 (8.1)
Chronic obstructive lung disease	4331 (11.0)	3970 (13.2)	3262 (12.7)	5039 (11.5)
Myocardial infarction	19 999 (50.7)	16 692 (55.4)	14 209 (55.2)	22 482 (51.2)
Left ventricular ejection fraction				
>60%	20 300 (51.4)	13 758 (45.6)	11 616 (45.1)	22 442 (51.2)
46–60%	12 672 (32.1)	10 072 (33.4)	8388 (32.6)	14 356 (32.7)
30–45%	5305 (13.4)	4897 (16.2)	4458 (17.3)	5744 (13.1)
<30%	1201 (3.0)	1419 (4.7)	1289 (5.0)	1331 (3.0)
Congestive heart failure	4071 (10.3)	4150 (13.8)	4027 (15.6)	4194 (9.6)
NYHA ≥3	6673 (16.9)	5435 (18.0)	5231 (20.3)	6877 (15.7)
Left main disease	10 070 (25.5)	8750 (29.0)	6164 (23.9)	12 656 (28.8)
Number of grafts	3.5 ± 1.0	3.1 ± 0.9	3.3 ± 1.0	3.3 ± 1.0
Number of diseased vessels	2.7 ± 0.5	2.7 ± 0.5	2.8 ± 0.5	2.7 ± 0.5
Single-vessel disease	864 (2.2)	389 (1.3)	268 (1.0)	985 (2.2)
Double-vessel disease	9149 (23.2)	6970 (23.1)	5182 (20.1)	10 937 (24.9)
Triple-vessel disease	29 334 (74.3)	22 623 (75.0)	20 194 (78.4)	31 763 (72.4)
Operative status				
Elective	25 071 (63.5)	18 049 (59.9)	16 205 (62.9)	26 915 (61.3)
Urgent	13 365 (33.9)	11 013 (36.5)	8852 (34.4)	15 526 (35.4)
Emergency	1025 (2.6)	1047 (3.4)	671 (2.6)	1401 (3.2)
Salvage	17 (0.0)	37 (0.1)	23 (0.1)	31 (0.0)
On-pump surgery	36 425 (92.3)	28 802 (95.5)	24 214 (94.0)	41 013 (93.5)
Minimally invasive	500 (1.3)	60 (0.2)	207 (0.1)	353 (0.8)

Data are presented as count (percentage) or value ± standard deviation.

MAG: multi-arterial grafting; NYHA: New York Heart Association score; SAG: single-arterial grafting.

A Cox proportional hazard model was applied to the time-to-event analysis estimating hazard ratios (HRs) and 95% confidence intervals (CIs) for long-term mortality in the matched diabetic and non-diabetic cohorts separately, with stratification on patient pairs. The influence of diabetes on the survival were assessed by using diabetes as an interaction term embedded in the Cox regression model. The proportionality assumption was verified with Schoenfeld residuals. Graphical visualization on the rate of death over time was achieved by Kaplan–Meier (KM) survival functions for the matched groups. The secondary short-term end points were compared via McNemar’s paired *t*-test.

Sensitivity analysis was conducted through (i) introducing an exact-match constraint on the number of grafts and (ii) additionally adjusting for the year of operation to ensure robustness of our conclusion.

The presence of significance was defined by a two-tailed *P*-value of <0.05. *P*-values were not adjusted for multiplicity, and findings from subgroup analyses should therefore be considered exploratory in nature. All statistical analyses were performed using R, version 4.0.5 (R Foundation for Statistical Computing, Vienna, Austria).

## RESULTS

**Figure 1:**
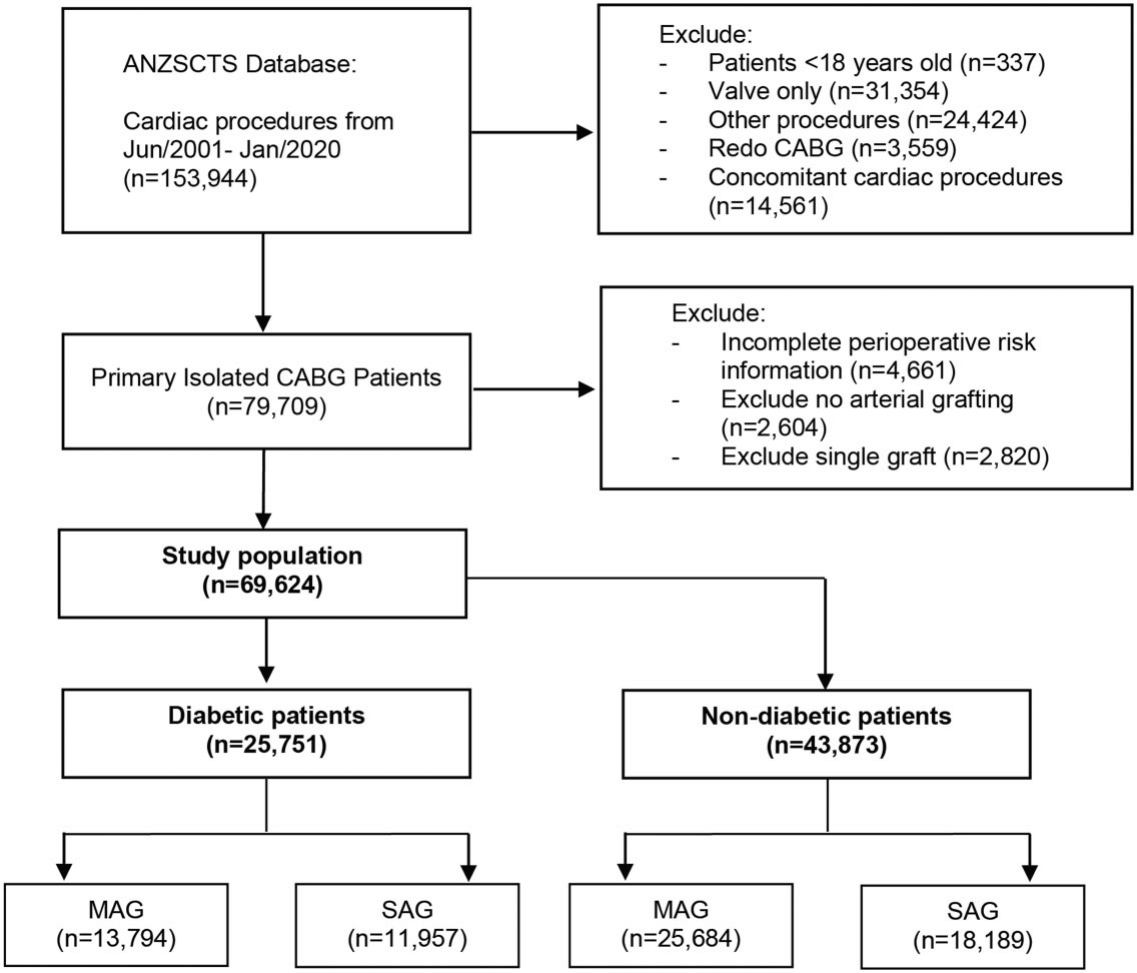
Selection process of eligible patients. ANZSCTS: Australian and New Zealand Society of Cardiac and Thoracic Surgeons; MAG: multiple arterial grafting; SAG: single-arterial grafting.

This study identified 69 624 eligible patients in total, which included 39 478 (56.7%) patients undergoing multiple arterial revascularizations and 25 751 (37.0%) patients with DM (Fig. 1). Among those with diabetes, 483 (1.9%) were not on any treatment, 3236 (12.6%) had dietary control, 14 349 (55.8%) were on oral treatment and 7683 (29.9%) were insulin-dependent. The proportional hazards assumption for the Cox regression model was verified with weighted residuals for all subsequent analyses. The median follow-up duration was 5.9 [interquartile range 3.2–9.6] years postoperatively. The mean follow-up index was 0.999 with an SD of 0.030.

In the unmatched cohort, compared to SAG, the MAG group was younger, more likely to be male and to have hypercholesterolaemia, but less likely to have other preoperative comorbidities including hypertension, DM, smoking history, dialysis, arrhythmia, MI and cerebrovascular event. Compared to non-diabetic patients, the diabetic group was younger and had higher female proportion and incidence of comorbidities with more severe coronary disease (Table 1).

### Primary outcome

#### Non-diabetics

**Figure 2:**
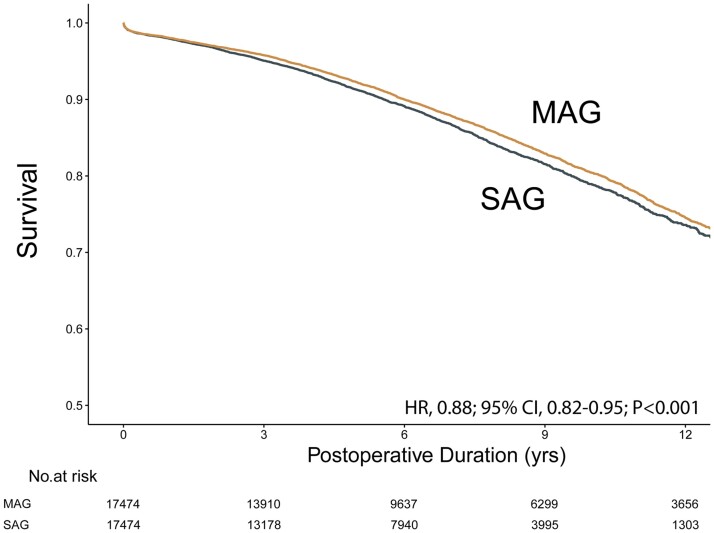
Kaplan–Meier survival curve for MAG versus SAG in the non-diabetic matched cohort. CI: confidence interval; HR: hazard ratio; MAG: multiple arterial grafting; SAG: single-arterial grafting.

The non-diabetic cohort generated 17 474 matched pairs, where the MAG group was associated with significantly improved long-term survival than the SAG group (HR, 0.88; 95% CI, 0.82–0.95; *P* < 0.001). The KM curve is shown in Fig. 2.

#### Diabetics

**Figure 3:**
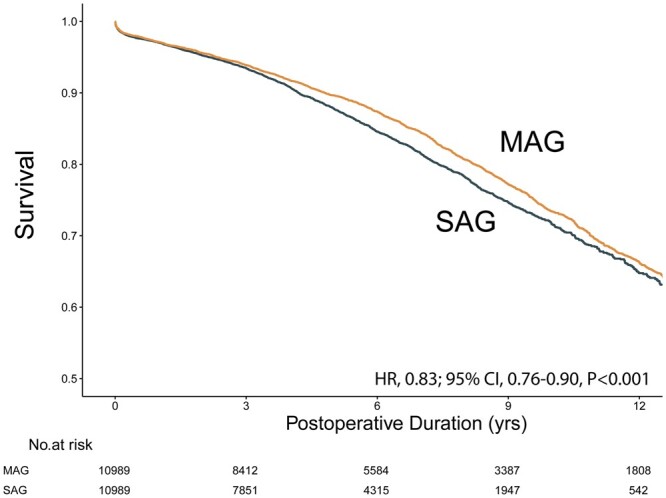
Kaplan–Meier survival curve for MAG versus SAG in the diabetic matched cohort. CI: confidence interval; HR: hazard ratio; MAG: multiple arterial grafting; SAG: single-arterial grafting.

In the diabetic population of 10 989 matched pairs, the MAG group again had superior survival than the SAG group (HR, 0.83; 95% CI, 0.76–0.90, *P* < 0.001). Interaction term analysis found insignificant subgroup interaction effect of diabetes on the survival difference (*P* = 0.55). The KM curve is shown in Fig. 3. Matching has reduced all standardized mean differences to below 10% except for the number of grafts used (SMD 0.11–0.12) (Table 2). The propensity score distribution plot confirmed high-quality matchings (Supplementary Material, Fig. S1).

**Table 2:** Patient demographics in diabetic and non-diabetic cohorts after matching

Variables	Diabetic population			Non-diabetic population		
	MAG group (*n* = 10 989)	SAG group (*n* = 10 989)	SMD	MAG group (*n* = 17 474)	SAG group (*n* = 17 474)	SMD
Age	65.6 ± 9.6	66.1 ± 9.8	0.06	66.3 ± 10.2	67.2 ± 10.1	0.09
Male	8530 (77.6)	8424 (76.7)	0.02	14 494 (82.9)	14 373 (82.3)	0.02
Body mass index	30.1 ± 5.5	30.1 ± 5.7	0	28.3 ± 4.8	28.2 ± 5.2	0.03
Creatinine (μmol/l)	102.9 ± 81.4	104.2 ± 78.4	0.02	93.1 ± 53.9	94.2 ± 63.6	0.02
Hypertension	9648 (87.8)	9722 (88.5)	0.02	13 125 (75.1)	13 404 (76.7)	0.04
Hypercholesterolemia	9602 (87.4)	9534 (86.8)	0.02	13 444 (76.9)	13 404 (76.7)	0.01
Diabetes mellitus	10 989 (100)	10 989 (100)	0	0	0	0
No treatment	202 (1.8)	209 (1.9)	0	0	0	0
Diet control	1416 (12.9)	1278 (11.6)	0.01	0	0	0
Oral therapy	6139 (55.9)	6142 (55.9)	0	0	0	0
Insulin dependent	3232 (29.4)	3360 (30.6)	0.03	0	0	0
Smoking history	7142 (65.0)	7158 (65.1)	0	11 233 (64.3)	11 259 (64.4)	0
Dialysis	154 (1.4)	162 (1.5)	0.01	102 (0.6)	109 (0.6)	0.01
Arrhythmia	957 (8.7)	1036 (9.4)	0.03	1541 (8.8)	1695 (9.7)	0.03
Cerebrovascular event	1285 (11.7)	1343 (12.2)	0.02	1547 (8.9)	1636 (9.4)	0.02
Peripheral vascular disease	1582 (14.4)	16 203 (14.6)	0.01	1453 (8.3)	1485 (8.5)	0.01
Chronic obstructive lung disease	1377 (12.5)	1453 (13.2)	0.02	2042 (11.7)	2173 (12.4)	0.02
Myocardial infarction	5982 (54.4)	6175 (56.2)	0.04	9013 (51.6)	9252 (52.9)	0.03
Left ventricular ejection fraction						
>60%	5032 (45.8)	4818 (43.8)	0.04	8835 (50.6)	8541 (48.9)	0.03
46–60%	3569 (32.5)	3645 (11.2)	0.01	5800 (33.2)	5861 (33.5)	0.01
30–45%	1869 (17.0)	1969 (17.9)	0.02	2309 (13.2)	2480 (14.2)	0.03
<30%	519 (4.7)	1969 (17.9)	0.02	530 (3.0)	592 (3.4)	0.02
Congestive heart failure	1671 (15.2)	1759 (20.4)	0.02	1697 (9.7)	1865 (10.7)	0.04
NYHA ≥3	2244 (20.4)	2246 (20.4)	0	2769 (15.8)	2752 (15.7)	0
Left main disease	2593 (23.6)	2730 (24.8)	0.03	5050 (28.9)	5331 (30.5)	0.04
Number of grafts	3.3 ± 0.9	3.2 ± 0.9	0.12	3.2 ± 0.9	3.1 ± 0.9	0.11
Number of diseased vessels	2.8 ± 0.5	2.8 ± 0.5	0	2.7 ± 0.5	2.7 ± 0.5	0
Single-vessel disease	111 (1.0)	89 (0.8)	0.02	354 (2.0)	297 (1.7)	0.02
Double-vessel disease	2227 (20.3)	2277 (20.7)	0.01	4386 (25.1)	4471 (25.6)	0.01
Triple-vessel disease	8612 (78.4)	8578 (78.1)	0.01	12 662 (72.5)	12 628 (72.3)	0
Operative status						
Elective	6988 (63.6)	6788 (61.8)	0.04	10 693 (61.2)	10 534 (50.3)	0.02
Urgent	3729 (33.9)	3898 (35.5)	0.03	6210 (35.5)	6314 (36.1)	0.01
Emergency	263 (2.4)	292 (2.7)	0.02	563 (3.2)	617 (3.5)	0.02
Salvage	9 (0.8)	11 (0.1)	0.01	8 (0.0)	9 (0.1)	0
On-pump surgery	10 388 (94.5)	10 485 (95.4)	0.03	16 529 (94.6)	16 662 (95.4)	0.03
Minimally invasive	41 (0.4)	26 (0.2)	0.01	60 (0.3)	34 (0.2)	0.01

Data are presented as count (percentage) or value ± standard deviation.

MAG: multi-arterial grafting; NYHA: New York Heart Association score; SAG: single-arterial grafting, SMD: standardized mean difference.

### Secondary outcomes

The incidence of 30-day MI was significantly higher after SAG than MAG in both diabetic (*P* = 0.029) and non-diabetic (*P* < 0.001) patients. Other short-term clinical outcomes remained similar between 2 procedures (Table 3).

**Table 3:** Paired McNemar’s test comparing short-term clinical outcomes between multi-arterial grafting and single-arterial grafting in diabetic and non-diabetic cohort

Short-term outcomes	Number of events (diabetic)			Number of events (non-diabetic)		
	MAG	SAG	*P*-Value	MAG	SAG	*P*-Value
30-Day mortality	129	205	0.407	172	248	0.720
30-Day readmission (overall)	301	282	0.452	360	319	0.121
Arrythmia	98	81	0.229	177	147	0.105
Chronic heart disease	68	65	0.862	77	60	0.172
Myocardial infarction	5	16	0.029	4	25	<0.001
Deep sternal infection	118	100	0.250	76	53	0.053
Recurrent angina	20	34	0.077	35	53	0.070

MAG: multi-arterial grafting; SAG: single-arterial grafting.

### Subgroup analysis of diabetic cohort

**Figure 4:**
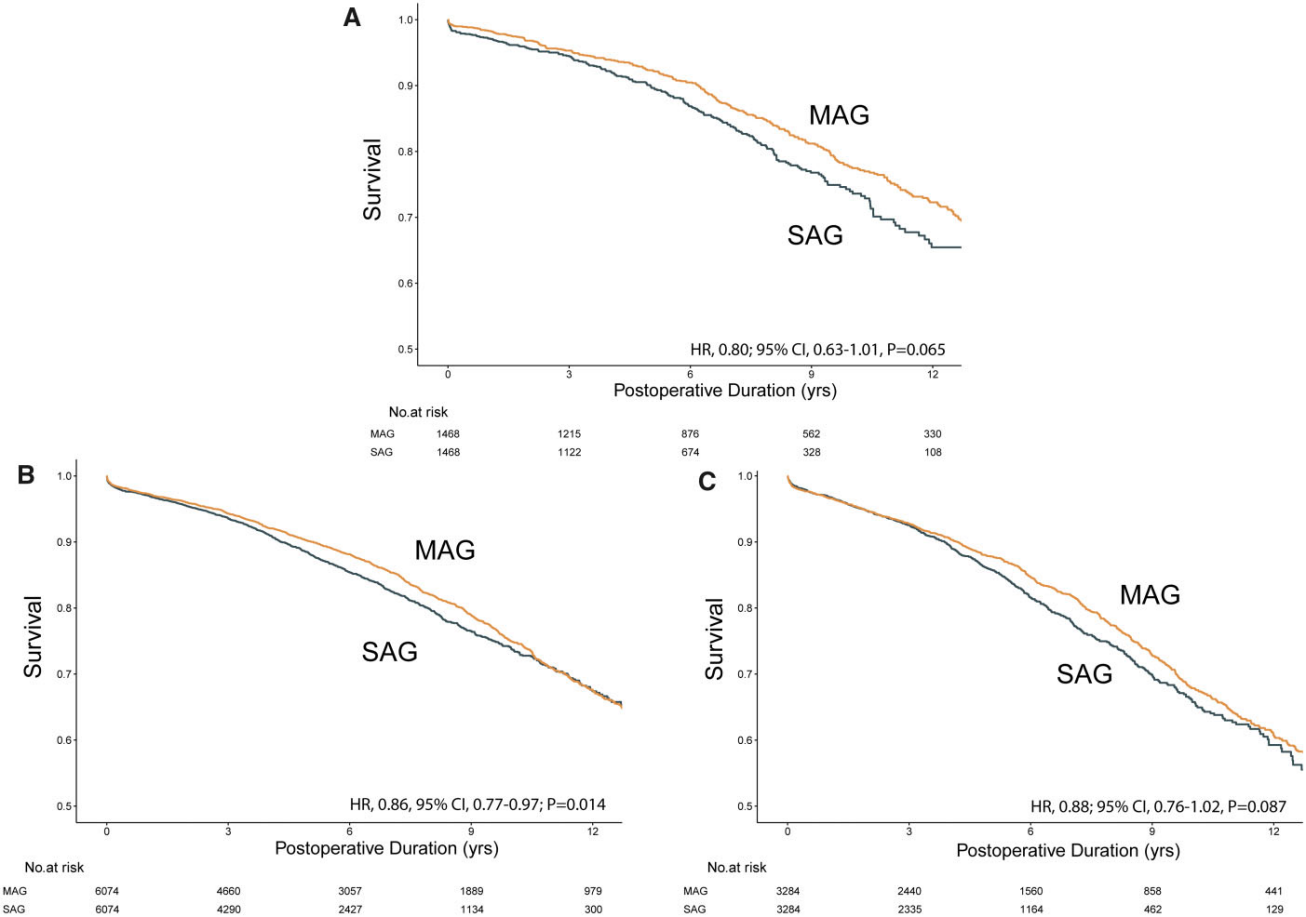
Kaplan–Meier curves showing all-cause mortality of MAG versus SAG according to diabetic subgroups: (**A**) patients without medical intervention, (**B**) patients with oral medication and (**C**) patients with insulin treatment. CI: confidence interval; HR: hazard ratio; MAG: multiple arterial grafting; SAG: single-arterial grafting.

After further stratification by the severity of DM, survival was observed at lowest rates in the insulin-dependent cohort (MAG, 60.7% [95% CI, 58.1–63.5%]; SAG, 59.3% [95% CI, 55.7–63.1%]) at 12 years postoperative. The MAG groups had significantly improved overall survival than SAG in patients with oral medication (HR, 0.86, 95% CI, 0.77–0.97; *P* = 0.014) whereas overall differences were not detected in diabetic patients without medical intervention (HR, 0.80; 95% CI, 0.63–1.01; *P* = 0.065) and with insulin treatment (HR, 0.88; 95% CI, 0.76–1.02, *P* = 0.087). The KM curves for subgroup mortality are shown in Fig. 4.

### Sensitivity analysis

After exactly matching for the number of grafts, MAG remained superior to SAG for overall long-term survival in both diabetic (HR, 0.85; 95% CI, 0.80–0.91; *P* < 0.001; 10,669 patient pairs) and non-diabetic patients (HR, 0.86; 95% CI, 0.81–0.91; *P* < 0.001; 17,255 patient pairs). PSM with additional adjustment for the year of operation also produced consistent results in diabetic (HR, 0.84; 95% CI, 0.79–0.90; *P* < 0.001; 10,328 patient pairs) and non-diabetic (HR, 0.88; 95% CI, 0.83–0.93; *P* < 0.001; 16,710 patient pairs) cohorts.

## DISCUSSION

This nation-wide study of 25 751 diabetic and 43 873 non-diabetic patients is the largest investigation in the literature to date comparing the impact of multiple arterial revascularization on long-term survival in diabetic and non-diabetic patients. The diabetic cohort had greater perioperative comorbidities and aggressive coronary disease than patients without diabetes. Nevertheless, MAG was associated with reduced all-cause mortality for both diabetic and non-diabetic patients. The interaction term analysis found insignificant subgroup effect from diabetes, but mortality rates numerically increased with diabetic severity. For insulin-dependent diabetes, the treatment effect remained similar to other diabetic treatment groups, but only with borderline significance. The incidence of 30-day MI was significantly higher in patients undergoing SAG regardless of diabetic status.

A novel finding of this study was that the survival benefit provided by MAG appears to be time-sensitive, which appears maximal between ∼6–9 years and declines after 9 years postoperatively. The mechanism remains unknown but could relate to the late graft failure of saphenous veins in MAG patients which results in an increased cumulative mortality effect despite a reduced number of SVG being at risk of failure compared to SAG.

The FREEDOM trial documented a strong survival benefit as well as a reduction in major cardiac and cerebrovascular events following CABG compared to drug-eluting stents [11]. Similarly, the SYNTAX Extended Survival Study with 12-year follow-up, demonstrated improved outcomes for MAG in diabetic patients. The updated ACC/AHA/SCAI guideline for bypass conduits proposed Class I recommendation for the use of radial artery as the supplementary conduit in preference to SVG for the second most important target [12], based on ease of use, superior durability and reduced adverse cardiac events and mortality [13–15]. However, MAG usage remains low in the world practice, and especially in diabetics [16].

Intense debate relating to the use of MAG in diabetic population continues because of discordant findings reported by prior investigations. Kunihara *et al.* [17] suggested indifferent long-term survival between LIMA-RA-T grafts and a combination of LIMA and sequential SVG. However, Yamaguchi *et al.* [18] reported significantly higher 12-year overall and major cardiac and cerebrovascular events-free survival in both diabetic (64.9% vs 58.8%; *P* = 0.041) and non-diabetic (71.4% vs 63.8%; *P* = 0.014) groups treated with MAG rather than SAG. The cumulative survival rates are consistent with our findings. A recent post hoc analysis of the Arterial Revascularization Trial [19] which documented significantly lower all-cause mortality at 10 years after MAG in patients with (HR, 0.68; 95% CI, 0.51–0.91); and without diabetes (HR, 0.83; 95% CI, 0.69–1.00). Importantly, most relevant studies only focused on a subset of MAG procedures (e.g. bilateral internal mammary technique) with limited generalizability and underpowered statistical inferences, which should be interpreted cautiously in the context of MAG.

MAG has a perceived increased risk of DSWI if BIMA is used. A meta-analysis of observational studies found that BIMA grafting increased DSWI by 62% [20]. The BIMA arm from the Arterial Revascularization Trial [21] also demonstrated higher incidence of DSWI (3.5% vs 1.9%; *P* = 0.005) and sternal reconstruction procedures (1.9% vs 0.6%; *P* = 0.002). In addition, its post hoc MAG analysis [19] on diabetic patients reported incrementally higher DSWI rates (7.9% vs 4.78%; *P* = 0.24). The influence of this complication on the long-term postoperative life expectancy after CABG is however uncertain. A recent meta-analysis of 24 studies at a mean follow-up duration of 3.5 years [22] reported increased overall mortality in patients with DSWI, whereas another 10-year study [23] suggested insignificant influence on risk of death in a general cardiac-surgical patient population. In this series, we stratified diabetic population by the type of medical management to comprehensively evaluate the subgroup interaction between DM and treatment effect of MAG versus SAG. The survival benefit of MAG appeared similar in all 3 diabetic cohorts. Significance was only detected in patients with oral medication but other groups consistently demonstrated a trend towards better MAG survival. In addition, our paired *t*-test did not detect any significant increase of DSWI in the MAG groups at least within 30-day follow-up. The use of MAG also appeared to have a protective effect against early MIs, but the mechanism would require further investigation.

### Limitations

The following limitations have to be considered during the interpretation of results. Although extensive PSM has been used to minimize selection bias inherent in retrospective observational studies, unmeasured confounders may limit analysis. Our database did not document harvest techniques, quality of conduits, preoperative coronary stenosis, target vessels or postoperative medical management. The number of grafts could not be perfectly matched (1–2% in excess to the 10% SMD recommendation) due to rigorous adjustment of 36 baseline covariates. The MAG groups had slightly higher average number of grafts (mean difference: 0.1 graft per patient) in both diabetic and non-diabetic populations, potentially introducing some bias. Nevertheless, a sensitivity analysis imposing exact matching on the number of grafts generated consistent results with the primary analysis.

## CONCLUSIONS

MAG was associated with significantly lower overall mortality compared to SAG in CABG patients with, or without, diabetes. A survival treatment effect was present in diabetic patients for MAG, though the insulin-dependent cohort failed to reach statistical significance.

## Supplementary Material

ezad091_Supplementary_Data

## Data Availability

The data underlying this article were provided by ANZSCTS by permission and cannot be shared publicly due to the privacy of individuals that participated in the study. The data will be shared on reasonable request to the corresponding author with permission of ANZSCTS.

## Notes

Presented at the 36th EACTS Annual Meeting, Milan, Italy, October 2022.

## ABBREVIATIONS

CI: Confidence intervals
CABG: Coronary artery bypass grafting
DSWI: Deep sternal wound infection
DM: Diabetes mellitus
HRs: Hazard ratios
KM: Kaplan–Meier
MAG: Multiple arterial grafting
MI: Myocardial infarction
PSM: Propensity score matching
SAG: Single-arterial grafting
SVG: Saphenous vein grafts
SDs: Standard deviations
