# Supplementary material for: Survival of multiple arterial grafting in diabetic populations: a 20-year national experience
Source: Eur J Cardiothorac Surg. 2023 Mar 16;63(6):ezad091. doi: 10.1093/ejcts/ezad091 (PMC10307942; doi:10.1093/ejcts/ezad091)

Online Supplementary Materials

Survival Of Multiple Arterial Grafting In Diabetic Populations: A Twenty-Year National Experience

Justin Ren,^a^ Colin Royse,^b,c^ David H. Tian,^a,d^ Aashray Gupta,^e,f^ Alistair Royse^a,b^

1. University of Melbourne, Melbourne, Australia
2. Royal Melbourne Hospital, Melbourne, Australia
3. Outcomes Research Consortium, Cleveland Clinic, Ohio, USA
4. Westmead Hospital, Sydney, Australia
5. University of Adelaide, Adelaide, Australia
6. Gold Coast University Hospital, Southport, Australia

​​**Index**

​Supplementary Figures S1

**Supplementary Figures S1.** Distribution of propensity scores before and after matching for diabetic and non-diabetic patients


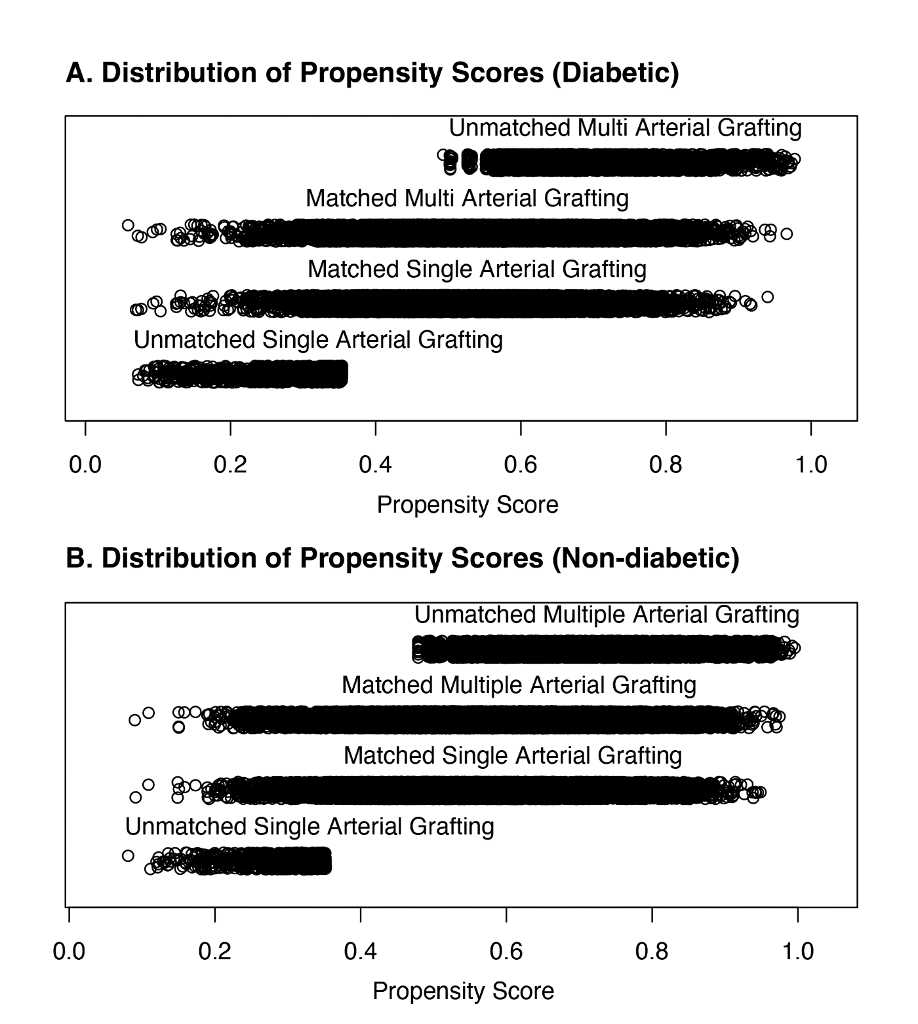

Supplement: ezad091_Supplementary_Data [file ezad091_supplementary_data.docx]
